# Systematic review and meta-analysis of association between plasminogen activator inhibitor-1 4G/5G polymorphism and recurrent pregnancy loss: an update

**DOI:** 10.1186/s12959-024-00612-9

**Published:** 2024-05-28

**Authors:** Mohaddese Maghsudlu, Zahra Noroozi, Elham Zokaei, Elahe Motevaseli

**Affiliations:** 1 Department of Medical Genetics, Faculty of Medicine, Tehran University of Medical Sciences, Tehran, Iran; 2 Department of Molecular Medicine, School of Advanced Technologies in Medicine, Tehran University of Medical Sciences, Tehran, Iran; 3 Department of Biology, Faculty of Sciences, Shahid Bahonar University of Kerman, Kerman, Iran

**Keywords:** Recurrent pregnancy loss, Plasminogen activator inhibitor-1, Thrombophilia, Meta-analysis

## Abstract

**Background:**

We conducted this systematic review and meta-analysis to better understand the association between rs1799762 *PAI-1* gene polymorphism and the risk of RPL.

**Methods:**

A systematic search for studies that assessed the association between *PAI-1 4G/5G* polymorphism and RPL risk published in search sources, PubMed/Medline, ISI Web of Knowledge, Scopus, and Google Scholar till January 2024 was conducted.

**Results:**

There were 23 case-control studies in total, with a high degree of statistical heterogeneity among them which indicated the need for subgroup analysis. We found a significant positive association between the risk of RPL and 4G/4G *PAI-1* (OR: 2.57; 95% CI: 1.69-3.90), likewise 4G/5G (OR: 2/02 95% CI: 1.39-2.92) and mixed genotype (4G/4G+4G/5G) (OR: 2.31 95% CI: 1.81-2.93). Considering the ethnicity, the 4G/4G polymorphism is significantly associated with Asian descent (OR: 2.10; CI: 1.65-2.69) while the strong association (OR: 6.47; CI: 3.23-12.97) observed in the Greater Middle East descent is not statistically significant (P=0.16). *PAI-1 4G/5G* polymorphism association with RPL was only significant in Greater Middle East descent (OR: 2.93; CI: 2.41-3.56), and mixed genotype was significantly associated with RPL in Asian (OR: 2.37; CI: 1.55-3.61), Greater Middle East (OR: 3.01; CI: 2.16-4.19), and European populations (OR: 1.38; CI: 0.91-2.10). The association between RPL and *PAI-1 4G/4G* was significant for RPLs both under 12 weeks (OR: 1.82; 95% CI: 1.34-2.47), and under 24 weeks (OR: 1.46; 95% CI: 1.11-1.92), while considering heterozygote form the association was only significant for RPLs under 24 weeks (OR: 1.91; 95% CI: 1.58-2.31). Regarding the mixed genotype, there is a significant positive association between PAI-1 and RPL for RPLs under 12 weeks (OR: 2.09; 95% CI: 1.49-2.93), and under 24 weeks (OR: 2.10; 95% CI: 1.52-2.92).

**Conclusions:**

Our findings indicate a significant association between the rs1799762 *PAI-1* polymorphism and the risk of RPL.

## Background

Miscarriage is described as the loss of a pregnancy before the fetus reaches viability. Recurrent pregnancy loss (RPL) is defined by the American Society for Reproductive Medicine (ASRM) and the European Society of Human Reproduction and Embryology (ESHRE) as the loss of two or more pregnancies before 20–24 weeks of gestation, including both embryonic and fetal losses [49]. Anatomical malformations, immunological illnesses, chromosomal errors and genetic polymorphisms, lifestyle variables, and thrombophilic gene polymorphisms have all been proposed as susceptibility factors that raise the likelihood of pregnancy loss in otherwise healthy women [4, 36]. On the other hand, routine clinical evaluations leave roughly half of the couples unidentified [44].

A delicate equilibrium between maternal coagulation and fibrinolysis is required for successful implantation and a healthy pregnancy [45]. Pregnancy itself is a hyper-coagulation state characterized by an increase in coagulants (factors VII, VIII, IX, X, XII, fibrinogen, and von Willebrand factor [vWF]), decreased anticoagulant factors (protein C and protein S), and diminished fibrinolytic activities because of hormonal modifications to prevent excessive maternal hemorrhage. However, RPL, intrauterine fetal growth restriction (IUGR), preeclampsia, and venous thromboembolism (VTE) have all been connected to hyper-coagulation conditions, including inherited or acquired thrombophilia [6, 29, 33]. Thrombophilia is a common cause of RPL, accounting for 40–50% of all instances. When comparing women with RPL to controls, Laude et al. discovered that levels of circulating procoagulant micro-particles were greater in cases with RPL [3]. Thrombophilia may occur in a syncytiotrophoblast invasion of the maternal blood arteries, resulting in micro-thrombosis at the implantation site, resulting in RPL and implantation failure [13].

Plasminogen activators are serine proteases that are involved in the conversion of plasminogen to plasmin. The human plasminogen activator inhibitor-1 (*PAI-1*) gene has nine exons and eight introns and is found on the long arm of chromosome 7. (12.2 Kb). During the process of trophoblast invasion, PAI-1 is a critical regulator that controls proteolysis and maternal tissue remodeling [12]. Greater transcription of the *PAI-1* gene is linked to homozygosity of the 4G allele of the *PAI-1* gene in the promoter region, resulting in increased gene expression. Individuals who are homozygous for the 4G allele have the highest levels of PAI-1 in their plasma, while heterozygote intermediates and 5G homozygotes have the lowest levels [44].

Several research has looked into the potential risk of RPL in patients with *PAI-1* 4G/5G polymorphism in recent years, but the results have been equivocal or conflicting [2, 8, 9, 17, 22, 24, 25, 38, 42]. This contradiction could be due to issues such as uncorrected multiple hypothesis testing, inadequate statistical power, publication biases, and ethnic inequalities. Previous meta-analyses [14, 15] based on published material have looked into the possibility of RPL susceptibility with the *PAI-1* 4G/5G polymorphism. Several more single-center investigations have recently been conducted [9, 21, 26, 31, 48]. To evaluate the existing totality of information on the risk of RPL with *PAI-1 4G/5G* polymorphism, we conducted this updated systematic review and meta-analysis utilizing stronger search parameters and a methodological quality analysis of the included studies and data.

## Materials and methods

### Search strategy

A comprehensive literature search regarding *PAI-1* mutations and recurrent pregnancy loss was performed through the major databases of PubMed/Medline, Scopus, ISI Web of Knowledge, and Google Scholar till 30^th^ January 2024 using keywords of (4G/5G OR PAI-1 OR “plasminogen activator inhibitor-1” OR Thrombophilia) AND (abortion OR miscarriage OR “pregnancy loss”). Moreover, we considered the reference lists of included articles in order to discover any further studies. Language and time restriction was not applied. Two reviewers independently screened the retrieved articles to assess if the study would meet the criteria for inclusion.

### Inclusion criteria

All titles of papers and their abstracts were attentively screened to distinguish their relevance. If the abstract represented that potential inclusion criteria were met or if the abstract did not provide enough information to ensure a decision, full texts were reviewed. The studies were qualified for inclusion if they indicated the following criteria: (1) being case-control studies, (2) evaluating the relationship between *PAI-1* and the risk of RPL, (3) defining RPL as two or more losses less than 24 weeks of gestation, (4) detecting mutation through DNA analysis techniques (PCR-RFLP, PCR and reverse hybridization, ARMS-PCR and, real-time PCR, sequencing), and (5) publications in which effect sizes were reported by rate or risk ratios (RRs) or odds ratios (ORs). In case of disagreement about the inclusion of a study between the authors, a third person (E.M) assessed the study in question and made the final decision.

### Exclusion criteria

The applied exclusion criteria were (1) case reports, letters to editor, animal studies, reviews, and meta-analyses, (2) those did not report ORs or RRs as effect size, (3) studies on other polymorphisms of thrombophilic genes or polymorphisms in *PAI-1* gene other than 4G/5G, (4) studies on some other adverse pregnancy outcomes apart from fetal loss, and (5) studies including participants with RPL of known cause or RPL in patients with underling disease.

### Data extraction

All information from studies was extracted independently by two reviewers using a pre-designed table (Table 1). The following data were extracted from each article (1) first author's familial name, year of publication, country, mean age of the participants, sample size (number of included cases/controls in each study), methodology used for polymorphism detection, comparison of the number of abortions in patient vs controls, the reported ORs or RRs with corresponding 95% Confidence Intervals (CIs) for heterozygous or/and homozygous women for *PAI-1* based on time of RPL. Again any disagreements between the reviewers were resolved by the principal investigator (E.M).

**Table 1:** Main characteristics of studies examining the association between 4G/5G *PAI-1* polymorphism and the risk of RPL

**First author (year)**	**Country**	**mean age of case/control**	**Number of cases/control**	**Mutation assessment**	**Time of RPL**	**OR (95%CI) 4G/4G**	**OR (95%CI) 4G/5G**	**OR (95%CI) 4G/4G+4G/5G**	**Comparison**	**ref**
Dossenbach-Glaninger et al. (2003)	Austria	35.6 /36.6	49/48	PCR/reverse hybridization	<12	_	_	2 (0/8-5/2)	≥2 RPL vs. no RPL	[10]
Guan et al. (2005)	china	NA	127/117	PCR-RFLP	<12	4/8 (2/23-10/35)	_	4/8 (2/23-10/35)	≥3 RPL vs. no RPL	[16]
Krause et al. (2005)	Germany	29/29	133/133	allele specific PCR	<24	1/04 (0/6-1/9)	_	1/04 (0/6-1/9)	≥3 RPL vs. no RPL	[27]*
Vora et al. (2008)	India	26/24	136/100	allele specific PCR	<12	1/8 (0/8-4)	_	1/8 (0/8-4)	≥2 RPL vs. no RPL	[47]*
Vora et al. (2008)	India	26/24	119/100	allele specific PCR	>12	3 (1/4-6/5)	_	3 (1/4-6/5)	≥2 RPL vs. no RPL	[47]
Vora et al. (2008)	India	Match	79/100	PCR-RFLP	<12	4/1 (1/8-9/2)	_	4/1 (1/8-9/2)	≥2 RPL vs. no RPL	[46]*
Vora et al. (2008)	India	Match	30/100	PCR-RFLP	>12	6/8 (2/6-17/9)	_	6/8 (2/6-17/9)	≥2 RPL vs. no RPL	[46]
Vora et al. (2008)	India	Match	89/100	PCR-RFLP	Early and Late	2/1 (0/9-4/8)	_	2/1 (0/9-4/8)	≥2 RPL vs. no RPL	[46]
Ivanov et al. (2010)	Bulgaria	NA	110/97	PCR-RFLP	<12	_	2/5 (1/15-5/45)	2/5 (1/15-5/45)	≥2 RPL vs. no RPL	[19]
Jeddi-Tehrani et al.(2011)	Iran	<35	100/100	PCR-RFLP	<24			1.71 (0/98-3/09)	≥2 RPL vs. no RPL	[20]
Aarabi et al. (2011)	Iran	32.5/32.9	63/114	PCR-RFLP	<24	8/2 (1/8-36/5)	_	8/2 (1/8-36/5)	≥2 RPL vs. no RPL	[1]
Idali et al. (2012)	Iran	30.1/30	106/100	ARMS-PCR	<24	_	5/3 (2/4-11/7)	5/3 (2/4-11/7)	≥3 RPL vs. no RPL	[18]
Poursadegh Zonouzi et al. (2013)	Iran	30.2/31.5	89/50	ARMS-PCR	<24	_	1/03 (0/49-2/22)	1/03 (0/49-2/22)	≥2 RPL vs. no RPL	[39]
Magdoud et al. (2013)	Tunisia	32.4/31.9	304/371	allele specific PCR	<24	_	2/78 (1/95-3/97)	2/78 (1/95-3/97)	≥3 RPL vs. no RPL	[34]
Jeon et al. (2013)	Korea	32.9/33.2	308/227	PCR-RFLP	<24	1/496 (0/89-2/5)	0/935 (0/57-1/53)	1/15 (0/72-1/83)	≥2 RPL vs. no RPL	[21]
Parveen et al. (2013)	India	NA	200/300	sequencing	<12	1/05 (0/63-1/72)	1/31 (0/86-2/01	1/22 (0/82-1/81	≥3 RPL vs. no RPL	[37]
Elmahgoub et al. (2014)	Egypt	28.5/29.1	120/100	PCR-RFLP	<12	3/52 (0/9-13/72)	_	3/52 (0/9-13/72)	≥3 RPL vs. no RPL	[11]
Elmahgoub et al. (2014)	Egypt	28.5/29.1	120/100	PCR-RFLP	<12	_	1/74 (0/98-3/1)	1/74 (0/98-3/1)	≥3 RPL vs. no RPL	[11]
Khosravi et al. (2014)	Iran	29.5/33	421/100	PCR-RFLP	<24	47/81 (6/52-350/65)	_	47/81 (6/52-350/65)	≥2 RPL vs. no RPL	[25]
Khosravi et al. (2014)	Iran	29.5/33	251/100	PCR-RFLP	<24	_	4/33 (2/64-7/10)	4/33 (2/64-7/10)	≥3 RPL vs. no RPL	[25]
Lino et al. (2015)	Brazil	30.3/40.2	106/98	sequencing	<12	0/82 (0/34-1/97)	1/62 (0/89-1/96)	1/39 (0/79-2/45)	≥3 RPL vs. no RPL	[31]
Shakarami et al. (2015)	Iran	NA	100/100	PCR-RFLP	<24	4.63 (1/55-13/84)	_	4.63 (1/55-13/84)	≥2 RPL vs. no RPL	[41]
Shakarami et al. (2015)	Iran	NA	100/100	PCR-RFLP	<24	_	1.36 (0/75-2/47)	1.36 (0/75-2/47)	≥2 RPL vs. no RPL	[41]
Kurzawinska et al. (2016)	Polish	30.1/29.4	152/100	PCR-RFLP	<24	0/91 (0/58-1/44)	_	0/91 (0/58-1/44)	≥2 RPL vs. no RPL	[28]
Kurzawinska et al. (2016)	Polish	30.1/29.4	152/100	PCR-RFLP	<24	_	1/09 (0/71-1/68)	1/09 (0/71-1/68)	≥2 RPL vs. no RPL	[28]
Salazar Garcia et al. (2016)	America	36.2/36.2	113/92	allele specific PCR	<24	_	_	1.32 (0/71-2/45)	≥2 RPL vs. no RPL	[40]
Chatzidimitriou et al. (2017)	Greek	35.3/35.1	48/27	PCR/reverse hybridization	<24	19/29 (2/42-153/87)	_	19/29 (2/42-153/87)	≥2 RPL vs. no RPL	[7]*
Bigdeli et al. (2018)	Iran	23/25.1	200/200	PCR-RFLP	>12	_	5/57 (3/62-8/58)	5/57 (3/62-8/58)	≥2 RPL vs. no RPL	[5]*
Jusić et al. (2018)	Bosnia	33.05/34.8	60/80	PCR-RFLP	<24	_	_	1.56 (0/79-3/07)	≥2 RPL vs. no RPL	[23]*
Miljanovic et al. (2023)	Montenegro	34.5/35.47	129/95	Allele Specific PCR	<24	_	_	2.92 (1.52-5.34)	≥2 RPL vs. no RPL	[35]*

### Statistical analysis

The analyses were conducted by the use of version 14^th^ of Stata software. For all obtained results, the statistical significance range was considered under 0.05. To calculate log OR and its standard errors (SEs), ORs and RRs with 95% CIs for odds of RPL were considered. Random effects model was used to calculate Pooled odds ratios (OR) and their 95% CIs, considering inter-study variability. A meta-analysis on the association between the risk of RPL and polymorphisms of *PAI-1 4G/5G* (heterozygote), *4G/4G* (homozygote), and *4G/5G+4G/4G* (mixed genotype) genotypes was performed. I-squared and Cochrane’s Q test were used to assess the heterogeneity between studies and it was considered significant when I^2^>50%. Funnel plot asymmetry was inspected visually to evaluate publication bias and statistical assessment of these plots was performed by Egger’s regression asymmetry tests. To investigate the extent to which results might depend on a specific study or group of studies, sensitivity analysis was carried out.

## Results

### Characteristics of included studies

Based on our search strategy overall 1352 articles on the association between *PAI-1 4G/5G* polymorphism and RPL were retrieved through comprehensive database searching. Through the screening and selection process, 1329 articles were excluded. Ultimately 30 effect sizes from 23 publications [1, 5, 7, 10, 11, 16, 18–21, 23, 25, 27, 28, 31, 34, 35, 37, 39–41, 46, 47] were considered eligible for the final meta-analysis, including 4284 RPL cases and 3549 controls. Table 1 presents the detailed data of the included publications.

For *PAI-1 4G/4G* polymorphism 2231 cases and 1961 controls were included [1, 7, 11, 16, 21, 25, 27, 28, 31, 37, 41, 46, 47], while for *PAI-1 4G/5G* polymorphism 2278 cases and 1846 controls [5, 11, 18, 19, 21, 25, 28, 31, 34, 37, 39, 41] and for *PAI-1 4G/4G+4G/5G* (mixed genotypes) 4284 cases and 3549 controls were considered [1, 7, 5, 10, 11, 16, 18–25, 27, 28, 31, 34, 35, 37, 39–41, 46, 47]. The complementary data about retrieved studies like the country where the study was conducted, number of cases and controls in each study, number of RPLs, time of RPLs, mean age of cases and controls, and the mutation assessment techniques are indicated in Table 1.

### Meta-analysis on *PAI-1* 4G/4G (homozygous) and RPL risk

**Fig. 1:**
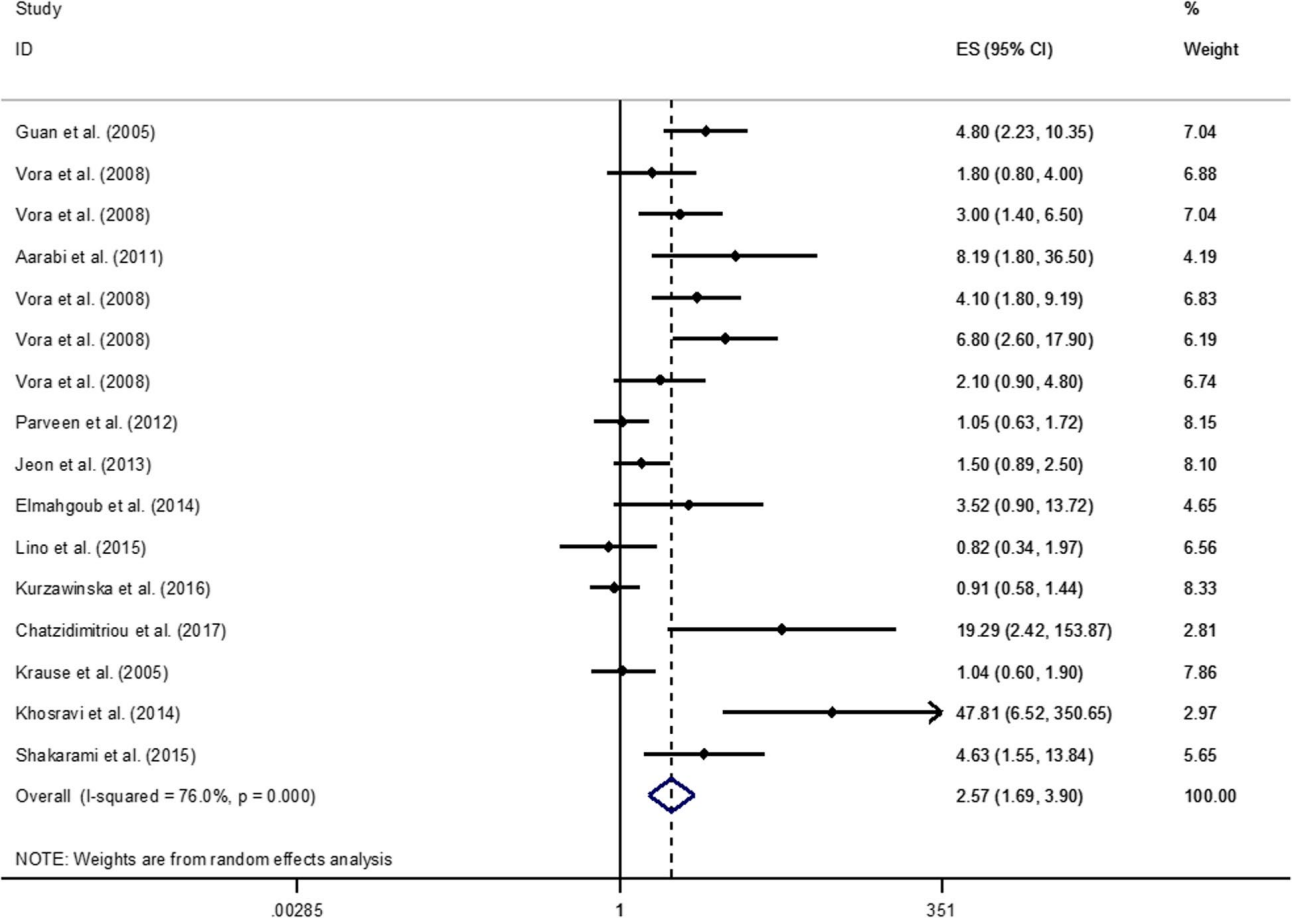
Association of homozygous *PAI-1* 4G/4G and risk of RPL

The results of the meta-analysis on homozygous *PAI-1 4G/4G* and the risk of RPL are shown in Fig. 1. Among the selected publications, 13 studies investigated the association between *PAI-1 4G/4G* polymorphism and risk of RPL. Each timing category for RPL in these studies was considered as a separate group; therefore, we derived 16 effect sizes from 13 studies.

Amongst, six effect sizes have been reported for the association of *PAI-1 4G/4G* and RPLs under 12 weeks [11, 16, 31, 37, 46, 47], two on the association of *PAI-1 4G/4G* and RPLs over 12 weeks [46, 47], seven effect sizes on the association of *PAI-1 4G/4G* RPLs under 24 weeks [1, 7, 21, 25, 27, 28, 41] and one effect size on this association and early-late RPL [47].

Combining 16 effect sizes derived from 13 case-control studies, depicted an overall 2.5-fold significant increased risk of RPL in homozygous women with PAI*-1 4G/4G* polymorphism (OR: 2.57; 95% CI: 1.69-3.90; *P*=0.00). The heterogeneity among studies was found to be significant (I^2^=76%, *P*<0.000). Therefore, we performed subgroup analysis based on time of RPL, race (was identified according to the location of the study, when it was not clearly stated), polymorphism assessment method, and number of losses to find any possible sources of the heterogeneity. The results of the subgroup analysis are shown in Table 2. Evidence of a significant publication bias was found (Egger’s test=0.00). In addition, sensitivity analysis indicated that no single study affected the findings. When the ratio comparing odds of homozygous form of *PAI-1 4G/4G* in the RPL and control groups were calculated with its 95% confidence interval (CI), a significant increase in the risk of recurrent miscarriage was observed with RPLs under 12 weeks (OR:1.82; 95% CI: 1.34-2.47), and under 24 weeks (OR: 1.46; 95% CI: 1.11-1.92), while it was not statistically significant in RPLs over 12 weeks (OR: 4.12; 95% CI: 2.26-7.51).

**Table 2:** Supplementary. Subgroup analysis for the association between PAI-1 4G/4G and the risk of RPL

**Variables**	**PAI-1 4G/4G** **OR (95%CI)**	**I**^**2**^	**P**_**between**_	**Number of Effect sizes**
**Race**				
Asian	2.10 (1.65-2.69)	69.1	0.002	8
Greater Middle Eastern	6.47 (3.23-12.97)	41.1	0.165	4
European	1.04 (0.73-1.48)	74.8	0.019	3
Latin American	0.82 (0.34-1.97)	-	-	1
**NO of miscarriage**				
≥2	2.09 (1.64-2.65)	76.7	0.000	11
≥3	1.38 (1.01-1.88)	74.0	0.004	5
**Mutation assessment**				
PCR-RFLP	1.90 (1.52-2.37)	79.6	0.000	11
PCR and reverse hybridization	19.29 (2.42-153.82)	-	-	1
Allele Specific PCR	1.59 (1.06-2.37)	58.4	0.091	3
Sequencing	0.82 (0.34-1.97)	-	-	1
**Time of RPL**				
≤12 weeks	1.82 (1.34-2.47)	73.1	0.002	6
≥12 weeks	4.12 (2.26-7.51)	40.0	0.193	2
≤24 weeks	1.46 (1.11-1.92)	81.6	0.000	7
Early-late	2.10 (0.91-4.85)	-	-	1

According to subgroup analysis based on race, the association of RPL and homozygous polymorphism (4G/4G) was higher than six folds in Greater Middle East (OR: 6.47; CI: 3.23-12.97) but it wasn’t statistically significant (*P*=0.16), while it was significant in Asian subgroup (OR: 2.10; CI: 1.65-2.69) and European population (OR: 1.04; CI: 0.73-1.48). The number of abortions was also significantly associated with *PAI-1 4G/4G* (*P*=0.00), for both recurrent miscarriages ≥2 (OR: 2.09; CI: 1.64-2.65) and recurrent miscarriages ≥3 (OR: 1.38; CI: 1.01-1.88).

In connection with the mutation assessment techniques used in the included studies, 4 different methods were used and PCR-RFLP was the most repetitive one. Differences in the accuracy and performance of these techniques can act as a source of heterogeneity.

### Meta-analysis on *PAI-1* 4G/5G (heterozygous) and RPL risk

**Fig. 2:**
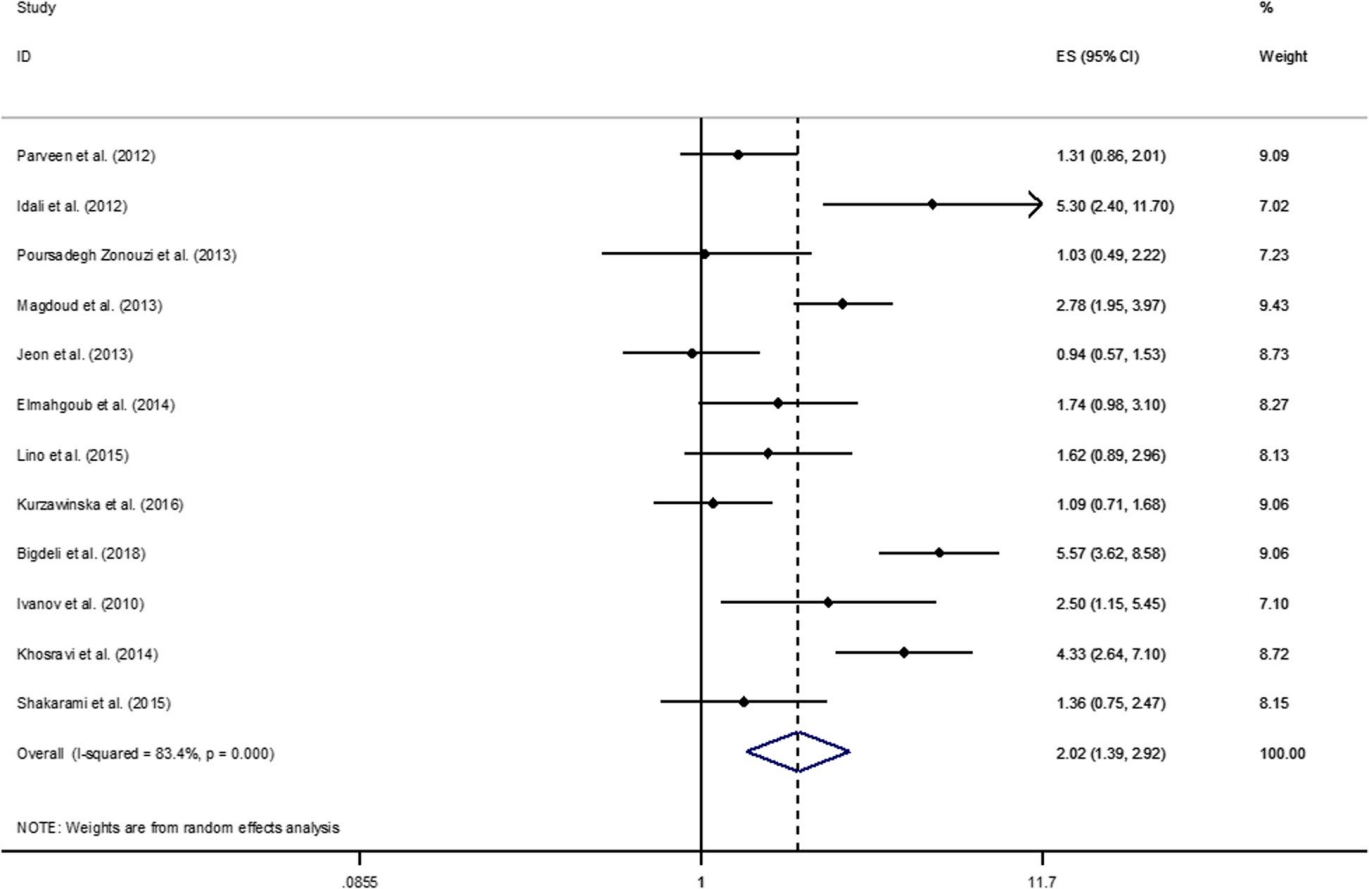
Association of heterozygous *PAI-1* 4G/5G and risk of RPL

The results of the meta-analysis on heterozygous *PAI-1 4G/5G* and the risk of RPL are shown in Fig. 2. The association between *PAI-1 4G/5G* polymorphism and risk of RPL was inquired among 12 publications of the selected studies. Considering the details, four effect sizes have been reported for the association of *PAI-1 4G/5G* and RPLs under 12 weeks [11, 19, 31, 37], one on the association of *PAI-1 4G/5G* and RPLs over 12 weeks [5], and seven effect sizes on the association of *PAI-1 4G/5G* RPLs under 24 weeks [18, 21, 25, 28, 34, 39, 41].

The analysis indicated an overall 2-fold significant increase in the risk of RPL for heterozygous women with *PAI-1 4G/5G* polymorphism (OR: 2.02; 95% CI: 1.39-2.92: *P*=0.00). Subgroup analysis has been performed considering the significant heterogeneity among studies (I^2^=83.4%, *P*<0.000), and the results are shown in Table 3. Significant publication bias was found (Egger’s test=0.805), moreover; sensitivity analysis confirmed that no single study had an effect on the findings. Comparing the heterozygous form of *PAI-1* in RPL and control groups, the results infer a significant increase in the risk of recurrent miscarriage with RPLs under 24 weeks (OR: 1.91; 95% CI: 1.58-2.31), but not in RPLs under 12 weeks (OR: 1.59; 95% CI: 1.20-2.10). In addition, comparing between different descent, the association of *PAI-1 4G/5G* polymorphism and RPL was significantly more than two folds in Greater Middle East (OR: 2.93; CI: 2.41-3.56) while it wasn’t significant in Asian (OR: 1.14; CI: 0.82-1.57) and European (OR: 1.32; CI: 0.91-1.93) populations. Considering the association between *PAI-1 4G/5G* polymorphism and the number of abortions, the association was statistically significant in both groups (*P*=0.00), albeit there was not a distinguishable difference between the two groups, for ≥2 RPLs (OR: 2.03; CI: 1.66-2.48) and for ≥3 RPLs (OR: 2.08; CI: 1.67-2.59).

**Table 3:** Supplementary. Subgroup analysis for the association between PAI-1 4G/5G and the risk of RPL

**Variables**	**PAI-1 4G/4G** **OR (95%CI)**	**I**^**2**^	**P**_**between**_	**Number of effect sizes**
**Race**				
Asian	1.14 (0.82-1.57)	3	0.310	2
Greater Middle Eastern	2.93 (2.41-3.56)	80.0	0.000	7
European	1.32 (0.91-1.93)	70.1	0.067	2
Latin American	1.62 (0.89-2.95)	-	-	1
**NO of miscarriage**				
≥2	2.03 (1.66-2.48)	88.6	0.000	7
≥3	2.08 (1.67-2.59)	70.4	0.009	5
**Mutation assessment**				
PCR-RFLP	1.93 (1.61-2.30)	86.9	0.000	8
ARMS-PCR	2.25 (1.30-3.88)	88.4	0.003	2
Allele-Specific PCR	2.78 (1.95-3.97)	-	-	1
Sequencing	1.62 (0.89-2.95)	-	-	1
**Time of RPL**				
≤12 weeks	1.59 (1.20-2.10)	0	0.532	4
≥12 weeks	5.57 (3.62-8.58)	-	-	1
≤24 weeks	1.91 (1.58-2.31)	84.8	0.000	7

### Meta-analysis on *PAI-1* 4G/4G+ 4G/5G (Mixed Genotype) and RPL risk:

**Fig. 3:**
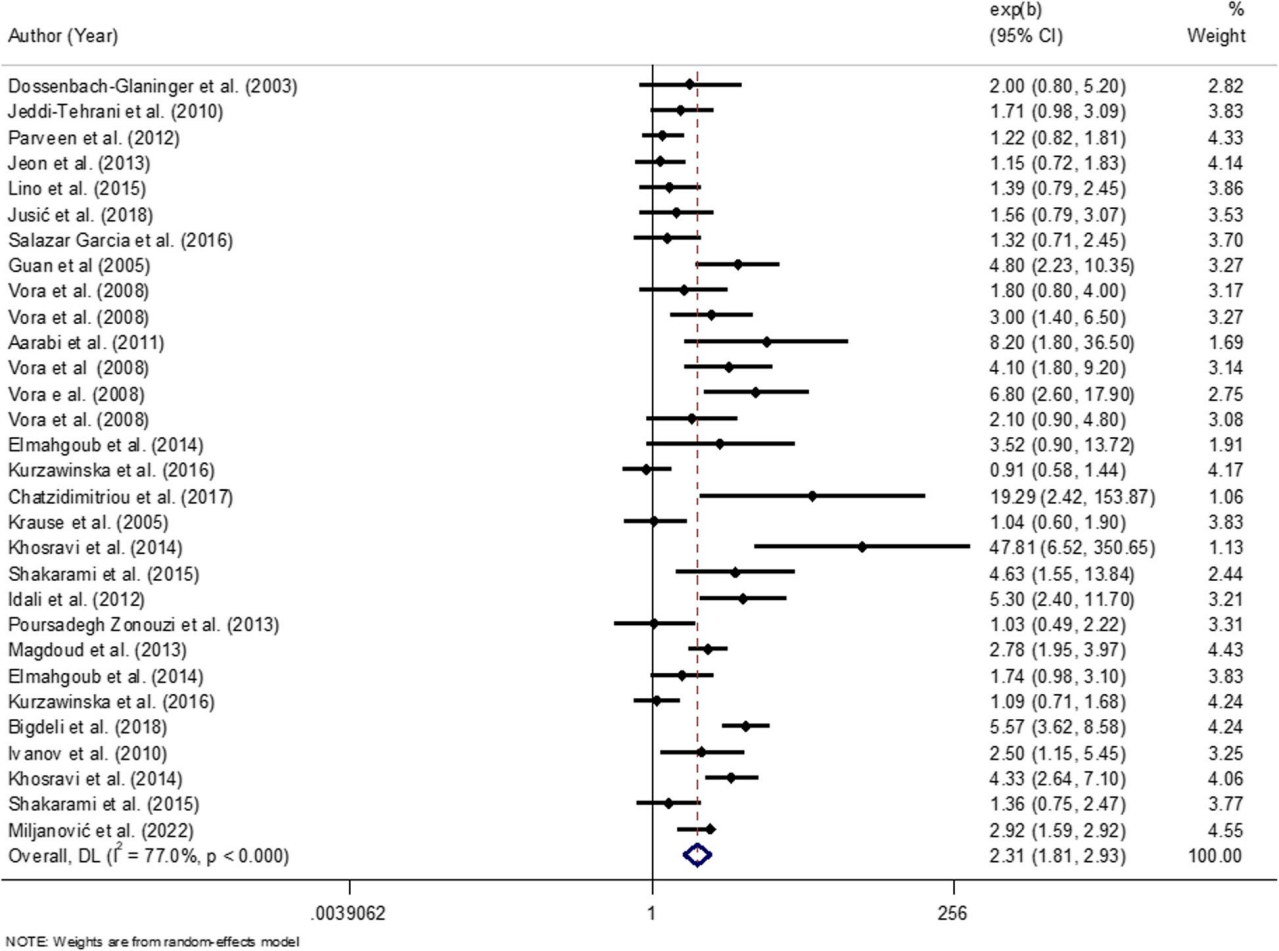
Association of *PAI-1* (4G/4G+4G/5G) and risk of RPL

The graphs related to the results of the meta-analysis on *PAI-1* mixed genotype and risk of RPL are provided in Fig. 3. Twenty-three studies out of the total included publications addressed the association between mixed genotype of *PAI-1* and the risk of RPL. Considering each different timing category for RPL in these studies as a separate group, we derived 30 effect sizes from 23 publications.

The association between mixed genotype and RPLs have been reported by nine effect sizes for under 12 weeks [10, 11, 16, 19, 31, 37, 46, 47], by three related effect sizes for over 12 weeks [5, 46, 47], by 17 effect sizes for under 24 weeks [1, 7, 18, 20, 21, 23, 25, 27, 28, 28, 34, 35, 39–41], and finally by one effect size on this association with early-late RPL [46, 47].

Considering the 30 size effects obtained from 23 studies, an overall 2.49-fold increased risk of RPL for mixed genotype (OR: 2.31; 95% CI: 1.81-2.93) was observed. The subgroup analysis was performed in accordance with the above, since the heterogeneity among studies found to be significant here as well (I^2^=79.5%, *P*<0.000).

The results of the subgroup analysis have been mentioned in Table 4. According to the results an evidence of a significant publication bias was found (Egger’s test=0.027) while, sensitivity analysis inferred that no single study affected the results. Analysis indicated that mixed genotype of *PAI-1* in the RPL compare to the control groups shows a significant increase in the risk of the recurrent miscarriage with RPLs under 12 weeks (OR: 2.09; 95% CI: 1.49-2.93), and under 24 weeks (OR: 2.10; 95% CI: 1.52-2.92), while it was not statistically significant in RPLs over 12 weeks (OR: 4.96; 95% CI: 3.32-7.40). In addition, comparing different populations, the association of mixed genotype and RPL was significantly around three folds in Greater Middle East (OR: 3.01; CI: 2.16-4.19) and it was also positive in Asian (OR: 2.37; CI: 1.55-3.61) and European (OR: 1.38; CI: 0.91-2.10) populations. Looking at the association between mixed genotype of *PAI-1* polymorphism and number of abortions, the association was statistically significant in both groups *(P*<0.00), although there was not a distinguishable difference between the two groups, for ≥2 RPLs (OR: 2.40; CI: 1.77-3.26) and for ≥3 RPLs (OR: 2.12; CI: 1.77-3.26).

**Table 4:** supplementary. Subgroup analysis for the association between PAI-1 mixed genotype and the risk of RPL

**Variables**	**PAI-1 4G/4G** **OR (95%CI)**	**I**^**2**^	**P**_**between**_	**Number of effect size**
**Race**				
Asian	2.37 (1.52-2.92)	69.7	0.000	9
Greater Middle Eastern	3.01 (2.16-4.19)	72.4	0.000	13
European	1.38 (0.91-2.10)	60.7	0.026	6
Latin American	1.36 (0.89-2.06)	-	-	2
**NO of miscarriage**				
≥2	2.40 (1.77-3.26)	78.5	0.000	22
≥3	2.12 (1.41-3.19)	74.5	0.000	8
**Mutation assessment**				
PCR-RFLP	2.49 (1.77-3.51)	81.0	0.000	19
PCR and reverse hybridization	2.93 (1.25-6.88)	73.7	0.051	2
Allele-Specific PCR	2.06 (1.43-2.95)	65.8	0.012	6
ARMS-PCR	2.33 (0.47-11.58)	88.4	0.003	2
Sequencing	1.39 (0.79-2.45)	-	-	1
**Time of RPL**				
≤12 weeks	2.09 (1.49-2.93)	51.0	0.038	9
≥12 weeks	4.96 (3.32-7.40)	14.2	0.312	3
≤24 weeks	2.10 (1.52-2.92)	80.02	0.000	17
Early-late	2.10 (0.91-4.85)	-	-	1

## Discussion

The results of this systematic review and meta-analysis of 22 case-control studies showed that *PAI-1 4G/5G* polymorphisms were significantly associated with RPL in both heterozygous and homozygous cases in addition for the mixed genotypes. The stratified analysis based on geographic region in contradiction with a former meta-analysis by Li et al. [32] revealed a significant association between the *PAI-1 4G/5G* polymorphism and the risk of RPL for all Greater Middle Eastern, Asians and Europeans populations. This association was stronger in Greater Middle East descent although it was not significant for homozygous cases and this stronger association might be due to higher number of studies that was conducted in these areas. The analysis of data based on time and the number of abortions showed that pregnant women carrying the polymorphism *PAI-1 4G/5G* were associated with an increased risk for RPL. The association was statistically significant in different groups (*P*=0.00), although there was not a distinguishable difference between the heterozygous, homozygous and mixed genotype groups.

Recurrent pregnancy loss is regarded as a difficult problem with many facets that is little understood. Growing evidences have accumulated over the years supporting the link between RPL and thrombophilic or hypofibrinolytic gene variations [2, 8, 9, 17, 22, 24, 25, 38, 42]. A hypofibrinolytic state can result from the *PAI-1-675 4G* variant, which is linked to gene overexpression. *PAI-1* is upregulated throughout implantation to alter trophoblast cells and stop hemorrhage during placentation. The most plausible explanation is that 4G alleles (including 4G/5G and 4G/4G) can increase *PAI-1* gene expression, preventing fibrin lysis that causes placental thrombosis and limiting trophoblastic migration through regulating proteolysis and maternal tissue modification, ultimately leading to abortion [30]. For women in the first trimester of pregnancy, high *PAI-1* expression is closely associated with hypofibrinolysis and thrombotic problems. Numerous case-control studies on the risk of RPL in women with the 4G/5G polymorphism of the *PAI-1* gene have been conducted, however the results have been inconsistent depending on the ethnic group and the study design [8, 23, 43]. Therefore, it is advised that pregnant women be screened for the *PAI-1-675* polymorphism, particularly those who experience RPL. If necessary, anticoagulant medication can be used during pregnancy to treat pregnant women who have the 4G allele and high levels of PAI-I.

Previous research on the relationship between *PAI-1* 4G/5G polymorphisms and the risk of RPL has produced conflicting result. Both the rising prevalence of these two polymorphisms in individuals with a history of RPL and the lack of a substantial correlation between these two polymorphisms and RPL have been noted. Therefore, combining the findings of several research can produce a more conclusive result than doing it in isolation. As a result, we carried out a meta-analysis and a systematic review to compile the findings of earlier research in this area. Our study revealed a strong correlation between RPL and *PAI-1* 4G/5G polymorphisms which concur with the findings of earlier meta-analyses. For instance while patients with homozygote 4G mutations were much more likely to experience RPL compared to healthy controls, Khosravi et al. [25] found a link between *PAI-1* and RPL and implantation failure (IF) in the Iranian population [25]. A study performed a systematic review and meta-analyses in which subgroup analysis showed a significantly elevated susceptibility to RPL in Asians, Caucasians, and Africans [17]. In concordance, our study showed that the *PAI-1* 4G/5G polymorphism probably provides a genetic contribution to the emergence of RPL. The findings might be used to create a theoretical framework for practical RPL prevention and treatment efforts.

On the other hand, Su et al. [44] published a systematic review and meta-analysis of 11 studies showing that high clinical heterogeneity existed among studies of *PAI-1* 4G/5G, and the aggregated data failed to confer higher susceptibility to idiopathic RPL in Caucasian and non-Caucasian patients [44]. A prior systematic review and meta-analysis found significant association between the *PAI-1* 4G/5G polymorphism and the risk of RPL under the recessive model (OR = 1.70, 95% CI = 1.21–2.38). However, in contrast to our findings no significant association between the *PAI-1 4G/5G* polymorphism and RPL was reported in this study [42].

The wide heterogeneity in clinical and methodological approaches of included studies was one of the obstacles in the way of this meta-analysis although we tried to handle this issue using sub group analysis. Considering the limitations of this study, we only included articles which had reported odd ratios thus some of the studies that had only reported frequencies were left out. In addition, we only looked at published studies, and also excluded some studies because they lacked sufficient data.

In summary, the significance of 4G/4G and 4G/5G polymorphisms as potential risk factors for RPL is highlighted by this meta-analysis. The results highlight the importance of routinely screening and analyzing *PAI-1* (4G/5G) polymorphism alterations in patients with RPL, particularly in Greater Middle East area.

## Data Availability

Not applicable.

## References

[1] Aarabi M, Memariani T, Arefi S, Aarabi M, Hantoosh Zadeh S, Akhondi MA, Modarressi MH (2011). Polymorphisms of plasminogen activator inhibitor-1, angiotensin converting enzyme and coagulation factor XIII genes in patients with recurrent spontaneous abortion. J Matern Fetal Neonatal Med.

[2] Adler G, Mahmutbegovic E, Valjevac A, Adler MA, Mahmutbegovic N, Safranow K, Czerska E, Pawinska-Matecka A, Ciechanowicz I (2018). Association between-675 ID, 4G/5G PAI-1 gene polymorphism and pregnancy loss: a systematic review. Acta Informatica Medica.

[3] Azem F, Many A, Yovel I, Amit A, Lessing JB (2004). Kupferminc MJ Increased rates of thrombophilia in women with repeated IVF failures. Hum Reprod.

[4] Baek KH, Lee EJ, Kim YS (2007). Recurrent pregnancy loss: the key potential mechanisms. Trends Mol Med.

[5] Bigdeli R, Younesi MR, Panahnejad E, Asgary V, Heidarzadeh S, Mazaheri H, Aligoudarzi SL (2018). Association between thrombophilia gene polymorphisms and recurrent pregnancy loss risk in the Iranian population. Syst Biol Reprod Med.

[6] Brenner B, Sarig G, Weiner Z, Younis J, Blumenfeld Z, Lanir N (1999). Thrombophilic polymorphisms are common in women with fetal loss without apparent cause. J Thromb Haemost.

[7] Chatzidimitriou M, Chatzidimitriou D, Mavridou M, Anetakis C, Chatzopoulou F, Lialiaris T, Mitka S (2017). Thrombophilic gene polymorphisms and recurrent pregnancy loss in Greek women. Int J Lab Hematol.

[8] Chen H, Nie S, Ming L (2015). Association between Plasminogen Activator Inhibitor-1 Gene Polymorphisms and Recurrent Pregnancy Loss: A Systematic Review and Meta-Analysis. Am J Reprod Immunol.

[9] Cho HY, Park HS, Ahn EH, Ko EJ, Park HW, Kim YR, Kim JH, Lee WS, Kim NK (2021). Association of Polymorphisms in Plasminogen Activator Inhibitor-1 (PAI-1), Tissue Plasminogen Activator (tPA), and Renin (REN) with Recurrent Pregnancy Loss in Korean Women. J Pers Med.

[10] Dossenbach-Glaninger A, van Trotsenburg M, Dossenbach M, Oberkanins C, Moritz A, Krugluger W, Huber J, Hopmeier P (2003). Plasminogen activator inhibitor 1 4G/5G polymorphism and coagulation factor XIII Val34Leu polymorphism: impaired fibrinolysis and early pregnancy loss. Clin Chem.

[11] Elmahgoub IR, Afify RA, Aal AA, El-Sherbiny WS (2014). Prevalence of coagulation factor XIII and plasminogen activator inhibitor-1 gene polymorphisms among Egyptian women suffering from unexplained primary recurrent miscarriage. J Reprod Immunol.

[12] Festa A, D’Agostino R, Rich SS, Jenny NS, Tracy RP, Haffner SM (2003). Promoter (4G/5G) plasminogen activator inhibitor-1 genotype and plasminogen activator inhibitor-1 levels in blacks, Hispanics, and non-Hispanic whites: the Insulin Resistance Atherosclerosis Study. Circulation.

[13] Floridon C, Nielsen O, Hølund B, Sweep F, Sunde L, Thomsen SG, Teisner B (2000). Does plasminogen activator inhibitor-1 (PAI-1) control trophoblast invasion? A study of fetal and maternal tissue in intrauterine, tubal and molar pregnancies. Placenta.

[14] Goodman C, Hur J, Goodman CS, Jeyendran RS, Coulam C (2009). Are polymorphisms in the ACE and PAI-1 genes associated with recurrent spontaneous miscarriages?. Am J Reprod Immunol.

[15] Goodman CS, Coulam CB, Jeyendran RS, Acosta VA, Roussev R (2006). Which thrombophilic gene mutations are risk factors for recurrent pregnancy loss?. Am J Reprod Immunol.

[16] Guan L, Du X, Wang J, Gao L, Wang R, Li H (2005). Association of genetic polymorphisms in plasminogen activator inhibitor-1 gene and 5, 10-methylenetetrahydrofolate reductase gene with recurrent early spontaneous abortion. Zhonghua yi xue yi Chuan xue za zhi= Zhonghua Yixue Yichuanxue Zazhi Chin J Med Genet.

[17] Huang Z, Tang W, Liang Z, Chen Q, Li M, Li Y, Lao S, Pan H, Huang L (2017). Plasminogen activator inhibitor-1 polymorphism confers a genetic contribution to the risk of recurrent spontaneous abortion: an updated meta-analysis. Reprod Sci.

[18] Idali F, Zareii S, Mohammad-Zadeh A, Reihany-Sabet F, Akbarzadeh-Pasha Z, Khorram-Khorshid HR, Zarnani AH, Jeddi-Tehrani M (2012). Plasminogen activator inhibitor 1 and methylenetetrahydrofolate reductase gene mutations in iranian women with polycystic ovary syndrome. Am J Reprod Immunol.

[19] Ivanov P, Komsa-Penkova R, Ivanov I, Konova E, Kovacheva K, Simeonova M, Tanchev S (2010). Plasminogen activator inhibitor type 1 activity in women with unexplained very early recurrent pregnancy loss. Akush Ginekol.

[20] Jeddi-Tehrani M, Torabi R, Zarnani AH, Mohammadzadeh A, Arefi S, Zeraati H, Akhondi MM, Chamani-Tabriz L, Idali F, Emami S, Zarei S (2011). Analysis of plasminogen activator inhibitor-1, integrin beta3, beta fibrinogen, and methylenetetrahydrofolate reductase polymorphisms in Iranian women with recurrent pregnancy loss. Am J Reprod Immunol.

[21] Jeon YJ, Kim YR, Lee BE, Choi YS, Kim JH, Shin JE, Rah H, Cha SH, Lee WS (2013). Genetic association of five plasminogen activator inhibitor-1 (PAI-1) polymorphisms and idiopathic recurrent pregnancy loss in Korean women. J Thromb Haemost.

[22] Joksic I, Mikovic Z, Filimonovic D, Munjas J, Karadzov ON, Egic A, Joksic G (2020). Combined presence of coagulation factor XIII V34L and plasminogen activator inhibitor 1 4G/5G gene polymorphisms significantly contribute to recurrent pregnancy loss in Serbian population. J Med Biochem.

[23] Jusić A, Balić D, Avdić A, Pođanin M, Balić A (2018). The association of factor V G1961A (factor V Leiden), prothrombin G20210A, MTHFR C677T and PAI-1 4G/5G polymorphisms with recurrent pregnancy loss in Bosnian women. Med Glas (Zenica).

[24] Kamali M, Hantoushzadeh S, Borna S, Neamatzadeh H, Mazaheri M, Noori-Shadkam M, Haghighi F (2018). Association between thrombophilic genes polymorphisms and recurrent pregnancy loss susceptibility in the Iranian population: a systematic review and meta-analysis. Iran Biomed J.

[25] Khosravi F, Zarei S, Ahmadvand N, Akbarzadeh-Pasha Z, Savadi E, Zarnani AH, Sadeghi MR, Jeddi-Tehrani M (2014). Association between plasminogen activator inhibitor 1 gene mutation and different subgroups of recurrent miscarriage and implantation failure. J Assist Reprod Genet.

[26] Kim JJ, Choi YM, Lee SK, Yang KM, Paik EC, Jeong HJ, Jun JK, Han AR, Hong MA (2014). The PAI-1 4G/5G and ACE I/D polymorphisms and risk of recurrent pregnancy loss: a case–control study. Am J Reprod Immunol.

[27] Krause M, Sonntag B, Klamroth R, Heinecke A, Scholz C, Langer C, Scharrer I, Greb RR, von Eckardstein A, Nowak-Göttl U (2005). Lipoprotein (a) and other prothrombotic risk factors in Caucasian women with unexplained recurrent miscarriage. J Thromb Haemost.

[28] Kurzawińska G, Barlik M, Drews K, Różycka A, Seremak-Mrozikiewicz A, Ożarowski M, Klejewski A, Czerny B (2016). Coexistence of ACE (I/D) and PAI-1 (4G/5G) gene variants in recurrent miscarriage in Polish population. Ginekol Pol.

[29] Kutteh, William H, and Douglas A Triplett. Thrombophilias and recurrent pregnancy loss. In Seminars in reproductive medicine. 2006 054-66. Copyright© 2006 by Thieme Medical Publishers. 10.1055/s-2006-931801 16418978

[30] Levin, Veronika, Rachel Booth, and Shahab Minassian. Bleeding Disorders and ART in Textbook of Assisted Reproduction 2020 Springer.

[31] Lino FL, Traina É, Barreto JA, Moron AF, Mattar R (2015). Thrombophilic mutations and polymorphisms, alone or in combination, and recurrent spontaneous abortion. Clin Appl Thromb Hemost.

[32] Li X, Liu Y, Zhang R, Tan J, Chen L, Liu Y (2015). Meta-analysis of the association between plasminogen activator inhibitor-1 4G/5G polymorphism and recurrent pregnancy loss. Med Sci Monit.

[33] Lussana Federico, Coppens Michiel, Cattaneo Marco, Middeldorp Saskia (2012). Pregnancy-related venous thromboembolism: risk and the effect of thromboprophylaxis. Thromb. Res..

[34] Magdoud K, Herbepin VG, Touraine R, Almawi WY, Mahjoub T (2013). Plasminogen activator inhibitor 1 4G/5G and− 844G/A variants in idiopathic recurrent pregnancy loss. Am J Reprod Immunol.

[35] Miljanović O, Ilić V, Teofilov S, Cikota-Aleksić B, Magić Z (2023). Polymorphisms of ACE and thrombophilic genes: risk for recurrent pregnancy loss. J Clin Pathol.

[36] Nguyen Ngoc N, Tran Ngoc Thao M, Trieu Tien S, Vu Tung S, Le H, Ho Sy H, Nguyen Thanh T, Trinh The S (2022). Evaluating the association between genetic polymorphisms related to homocysteine metabolism and unexplained recurrent pregnancy loss in women. Appl Clin Genet.

[37] Parveen Farah, Tuteja Moni, Agrawal Suraksha (2013). Polymorphisms in MTHFR, MTHFD, and PAI-1 and recurrent miscarriage among North Indian women. Arch Gynecol Obstet.

[38] Pereza Nina, Ostojić Saša, Kapović Miljenko, Peterlin Borut (2017). Systematic review and meta-analysis of genetic association studies in idiopathic recurrent spontaneous abortion. Fertil Steril.

[39] Poursadegh Zonouzi A, Chaparzadeh N, Ghorbian S, Sadaghiani MM, Farzadi L, Ghasemzadeh A, Kafshdooz T, Sakhinia M, Sakhinia E (2013). The association between thrombophilic gene mutations and recurrent pregnancy loss. J Assist Reprod Genet.

[40] Salazar Garcia MD, Sung N, Mullenix TM, Dambaeva S, Beaman K, Gilman-Sachs A, Kwak-Kim J (2016). Plasminogen Activator Inhibitor-1 4G/5G Polymorphism is Associated with Reproductive Failure: Metabolic, Hormonal, and Immune Profiles. Am J Reprod Immunol.

[41] Shakarami F, Akbari MT, Karizi SZ (2015). Association of plasminogen activator inhibitor-1 and angiotensin converting enzyme polymorphisms with recurrent pregnancy loss in Iranian women. Iran J Reprod Med.

[42] Shi X, Xie X, Jia Y, Li S (2017). Maternal genetic polymorphisms and unexplained recurrent miscarriage: a systematic review and meta-analysis. Clin Genet.

[43] Sosic GM, Sretenovic S, Radivojevic D, Jovic N, Varjacic M. The impact of the gene variants FV Leiden, FII G20210A, MTHFR C677T and PAI-1 4G/5G on pregnancy loss in women from Central Serbia. Serbian J Exp Clin Res. 2020:21(1):19–25.

[44] Su MT, Lin SH, Chen YC, Kuo PL (2013). Genetic association studies of ACE and PAI-1 genes in women with recurrent pregnancy loss Thromb. Haemost.

[45] Thornton P, Douglas J (2010). Coagulation in pregnancy. Best Pract Res Clin Obstet Gynaecol.

[46] Vora S, Shetty S, Ghosh K (2008). Thrombophilic dimension of recurrent fetal loss in Indian patients. Blood Coagul Fibrinolysis.

[47] Vora S, Shetty S, Salvi V, Satoskar P, Ghosh K. Thrombophilia and unexplained pregnancy loss in Indian patients. Natl Med J India. 2008;21:116–9. 19004141

[48] Wolf CE, Haubelt H, Pauer HU, Hinney B, Krome-Cesar C, Legler TJ, Hellstern P, Emons G, Zoll B, Köhler M (2003). Recurrent pregnancy loss and its relation to FV Leiden, FII G20210A and polymorphisms of plasminogen activator and plasminogen activator inhibitor. Pathophysiol Haemos Thromb.

[49] Youssef A, Vermeulen N, Lashley EL, Goddijn M, van der Hoorn ML (2019). Comparison and appraisal of (inter) national recurrent pregnancy loss guidelines. Reprod Biomed Online.

